# The Brain’s Dorsal Route for Speech Represents Word Meaning: Evidence from Gesture

**DOI:** 10.1371/journal.pone.0046108

**Published:** 2012-09-26

**Authors:** Goulven Josse, Sabine Joseph, Eric Bertasi, Anne-Lise Giraud

**Affiliations:** 1 Institut du Cerveau et de la Moëlle épinière, Hôpital de la Pitié-Salpêtrière, Paris, France; 2 Cognitive Neurology Group, Institute of Cognitive Neuroscience, University College London, London, United Kingdom; 3 Centre de NeuroImagerie de Recherche (CENIR), Centre de Recherche de l’Institut du Cerveau et de la Moëlle, Université Pierre et Marie Curie - INSERM UMRS 975 - CNRS UMR 7225, Hôpital de la Pitié-Salpêtrière, Paris, France; 4 Laboratoire de Neurosciences Cognitives - INSERM U960, Département d’Etudes Cognitives, Ecole Normale Supérieure, Paris, France; University Of Cambridge, United Kingdom

## Abstract

The dual-route model of speech processing includes a dorsal stream that maps auditory to motor features at the sublexical level rather than at the lexico-semantic level. However, the literature on gesture is an invitation to revise this model because it suggests that the premotor cortex of the dorsal route is a major site of lexico-semantic interaction. Here we investigated lexico-semantic mapping using word-gesture pairs that were either congruent or incongruent. Using fMRI-adaptation in 28 subjects, we found that temporo-parietal and premotor activity during auditory processing of single action words was modulated by the prior audiovisual context in which the words had been repeated. The BOLD signal was suppressed following repetition of the auditory word alone, and further suppressed following repetition of the word accompanied by a congruent gesture (e.g. [“grasp” + grasping gesture]). Conversely, repetition suppression was not observed when the same action word was accompanied by an incongruent gesture (e.g. [“grasp” + sprinkle]). We propose a simple model to explain these results: auditory and visual information converge onto premotor cortex where it is represented in a comparable format to determine (in)congruence between speech and gesture. This ability of the dorsal route to detect audiovisual semantic (in)congruence suggests that its function is not restricted to the sublexical level.

## Introduction

What we know of human brain function at a macro-anatomical level includes a couple of relatively well-accepted distinctions. In the communication domain, the left hemisphere is thought to be specialized for language processing relative to the right hemisphere [1], [2]. In the visual domain, a ventral stream of information processing is thought to be specialized in object recognition (“*What is it*?”), whereas a dorsal stream specializes in spatial processing (“*Where is it*?”) [3]. More recently, it was proposed that the auditory system may be similarly divided into dorsal and ventral streams [4]. Again, the questions “*What?*” and “*Where?*” were used as a metaphor to emphasize the major differences between ventral and dorsal streams in the perceptual domain. However, coincidentally or not, these questions almost obviously suggest a link to language. Building on this, Hickok and Poeppel have argued that not only the left-right, but also the ventral-dorsal distinction could be useful to understand how the brain processes language [5], [6]. Their dual-route model is actually more about *auditory speech* than about language which can also involve the visual modality through reading, visually perceived mouth movements, sign language or (as we will see here) gesture. In their own dual-route model, Hickok and Poeppel have proposed that, first, a bilateral temporal network (the ventral route) maps word to meaning at the lexico-semantic level, thus allowing for word comprehension (“*What does the sound I heard mean?*”). Second, a left-lateralized temporo-parieto-frontal network maps auditory-to-motor word features at the sublexical level. This may seem at odds with the “*Where*?” question. In fact, answering “*Where is the object?*” is a pre-requisite to *acting* on the object by grasping it and manipulating it using both the sensory (visual or auditory) system and the motor system. In other words, the dorsal route is a *sensori-motor* pathway [7]. In a context where objects are words, this dorsal route could thus enable speech parsing based on articulatory movements [8], as well as speech repetition and, therefore, learning to speak [5], [6]. This dual-route model is supported by many behavioral and neuroimaging studies [5], [9], [10]. However, it is also challenged by evidence that the premotor cortex of the dorsal route is involved in lexico-semantic processing (“*What?*”) in addition to sublexical processing. For instance, premotor activity is thought to underlie action words comprehension [11], [12]. Premotor activity has also been associated with the perception of gesture that carries meaning alongside speech [13]. In this article, we argue that the dorsal route serves to map auditory speech not only onto articulatory representations, but also onto gestural representations corresponding to speech-associated hand gesture. Since gesture carries meaning, the auditory-motor mapping function of the dorsal route necessarily engages semantic representations. Importantly, due to repeated associations between words and gesture, these gestural representations are engaged even when words are perceived or produced alone.

Our proposal that the (pre)motor representations of words and gesture overlap is based on several lines of evidence. Elegantly defined as “visible action as utterance” [14], speech-associated gesture has been described as an integral part of language [15]. Gesture is associated with speech in terms of meaning and the two are tightly synchronized. There is evidence that gesturing improves lexical retrieval in both normal subjects and Broca’s aphasics [16], [17], [18]. At the neuronal level, there are reports suggesting that hand and mouth representations could overlap in (pre)motor cortex [19], [20], [21], [22]. For instance, in people speaking while grasping an object at the same time, the size of the grasped object predicts the size of mouth openings [19]. This is the case despite the fact that the grasping task has nothing to do with the act of speaking other than both are taking place simultaneously. We can therefore suppose that gesture affects speech to an even greater extent when the two are congruent, and that the overlap between their neuronal representations is also greater.

We further propose that the overlap between word and gesture representations in premotor cortex can explain some previously reported neuroimaging findings. A few studies have investigated the neural correlates of speech comprehension in a context where visually meaningful, iconic gesture could potentially modulate speech processing. In order to control for biological motion, most of these studies used a control condition where speech was presented with either incongruent gesture, or meaningless hand movements (we will refer to both cases as incongruence). These studies using functional magnetic resonance imaging (fMRI) consistently reported that incongruence was associated with increased activity relative to a similar condition where speech and gesture were congruent, whereas congruence was not associated with any specific activity relative to incongruence [23], [24], [25], [26]. We reasoned that this might be the case because the overlap between the neuronal population representing the word and the population representing gesture is less extensive when word and gesture are incongruent: in this case more neurons are activated and, therefore, a larger BOLD signal is observed.

Alternatively, it has been proposed that increased semantic load in incongruent conditions, or after-effects unrelated to the process of comparing speech and gesture (such as inhibition), could explain increased activation [23], [25]. However, activation studies have so far only provided information at the macro-anatomical level that leaves both this question, and mechanisms of speech and gesture integration at the neuronal level, unaddressed.

To test our neuronal model of overlapping word and gesture representation, we use repetition effects on the BOLD signal. Stimulus repetition is usually associated with a decrease in both neuronal activity and BOLD signal [27], [28], [29]). Accordingly, activation of a neuronal population by a given word should decrease when the word is repeated. We hypothesize that many neurons in this neuronal population are also activated by any congruent gesture. Therefore, the combined presentation of congruent word and gesture should enhance the repetition suppression effect. To the contrary, if a word and a gesture are incongruent, each will target different neurons, and therefore the suppression effect related to word repetition will not be stronger when an incongruent gesture is repeated alongside the word. Our findings confirm our hypothesis that the dorsal route for speech is a major site of audiovisual integration and semantic processing.

**Figure 1:**
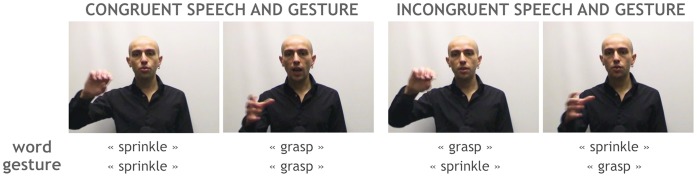
Stimuli. Stillframes of video stimuli from the two audiovisual conditions where Speech and Gesture were either Congruent (SGC) or Incongruent (SGI). In the two corresponding speech-only conditions (respectively Sc and Si), videos were shown with a black mask macthing the background, so that subjets only perceived the audio from the video files.

## Materials and Methods

### Subjects

Twenty-eight subjects participated in an fMRI experiment (13 females, age range [20 – 49 years], mean 26, standard deviation 7). All participants were right handed, according to the Edinburgh questionnaire (laterality index range: [+41 – +100], median 88) [30]. All participants were native French speakers. All were free of any psychiatric or neurological abnormalities, had normal or corrected-to-normal vision and reported no hearing impairments. The study was approved of by the local Ethics Committee and all 28 participants gave written informed consent to take part in the study.

**Figure 2:**
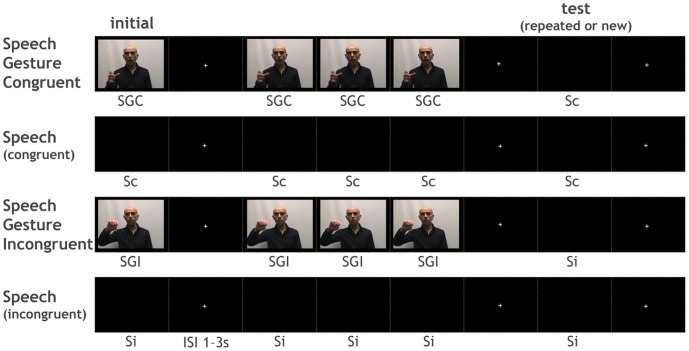
Design. Example of a trial in each of the 4 audio(visual) conditions. SGC: Congruent Speech and Gesture, Sc: audio corresponding to SGC, SGI: Incongruent Speech and Gesture, Si: audio corresponding to SGI, ISI: Inter-Stimuli Interval.

### Stimuli

All videos showed the same male actor uttering an action verb and simultaneously performing an iconic gesture (Figure 1 - the actor has given written informed consent to publication of his photograph). Before recording, 40 action verbs were selected based on their ability to suggest a related gesture to the actor. The list was copied and randomized in order to make 40 pairs of action verbs. Videos were then recorded pair by pair, starting with a congruent verb/gesture utterance. The gesture was performed as naturally as possible by the actor, without rehearsal or attempts on the actor’s part to improve the iconicity of the gesture. The actor subsequently reproduced the same gesture with ease while uttering the other action verb of the pair.

**Figure 3:**
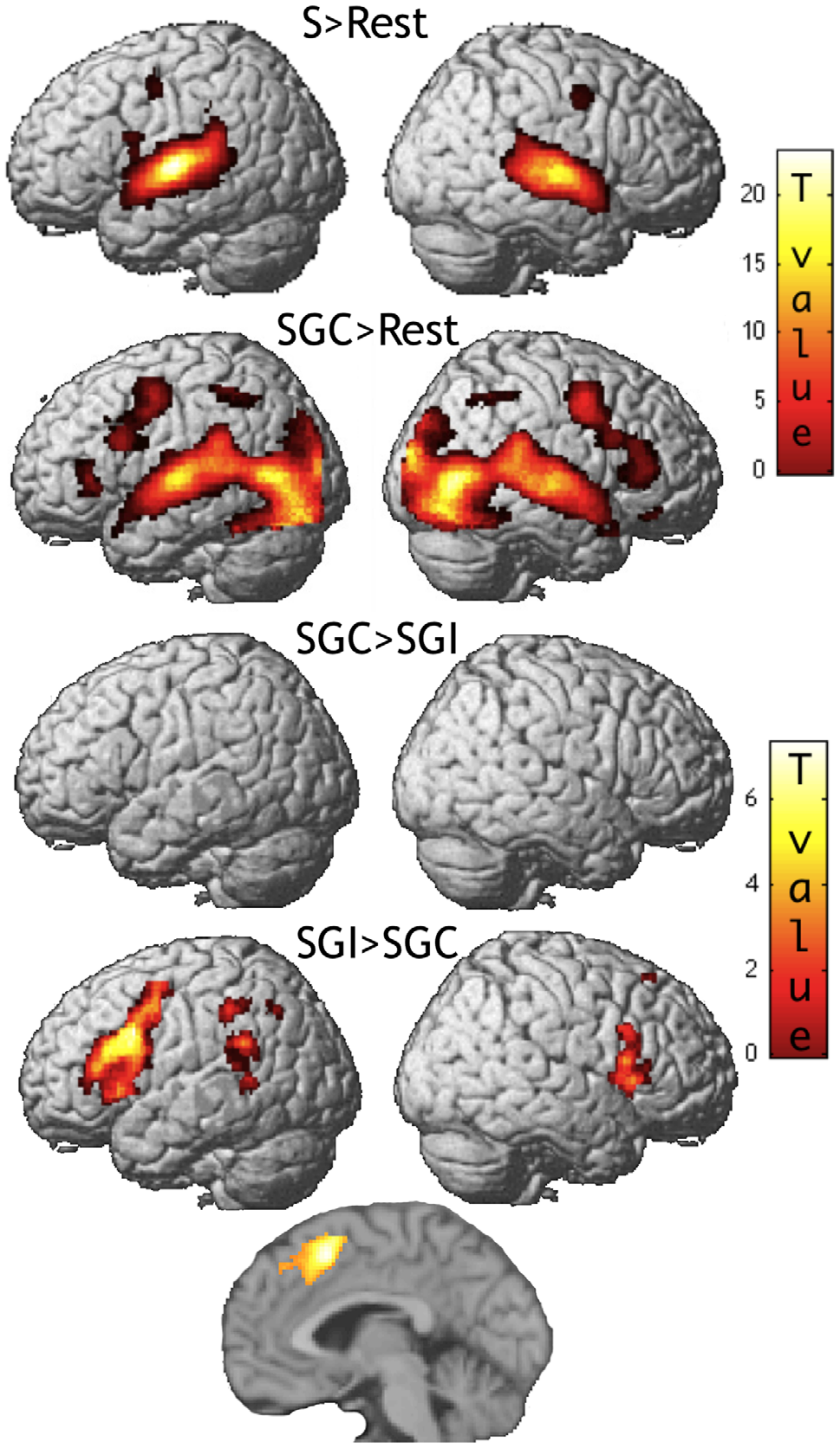
Initial Phase. SPM renderings with one medial section to show SGI>SGC activation in the supplementary motor area. Maps were thresholded at p<0.05 corrected for multiple comparisons either in terms of height or spatial extent. The right precentral cluster in S>Rest, approaching significance, is also shown. See Table 1 for details. S: Speech (average of Si and Sc between which no difference was found), SGC: Congruent Speech and Gesture, SGI: Incongruent Speech and Gesture.

**Table 1:** Initial phase.

Initial Sc > Rest				
Anatomical Location	x	y	z	Z-score
Left superior temporal gyrus	59	−15	6	Inf
Right superior temporal gyrus	57	−11	5	Inf
Right precentral gyrus	54	−1	47	5.5
Left precentral gyrus	−49	−10	48	4.5
**Initial SGC > Rest**				
Right anterior occipital sulcus	51	−68	2	Inf
Right precentral gyrus	50	1	51	Inf
Left intraparietal sulcus	−31	−51	50	7.8
Right postcentral sulcus	28	−36	51	6.8
Left central sulcus	−47	−6	55	6.6
Left inferior frontal gyrus(pars triangularis)	−36	31	1	4.7
**Initial SGI > SGC**				
Left supplementary motor area	−5	16	49	6.9
Left precentral/inferior frontal sulcus	−46	12	29	6.6
Right pars opercularis	47	14	15	5.5
Left supramarginal gyrus	−51	−48	25	5.2
Left intraparietal sulcus	−27	−61	40	4.8

All effects were significant after correcting for multiple comparisons across the whole brain, either in terms of intensity or spatial extent. Coordinates are in the template space of the Montreal Neurological Institute (transformed from the symmetrical template space used for normalization). Sc: Speech  =  audio corresponding to SGC, SGC: Congruent Speech and Gesture, SGI: Incongruent Speech and Gesture.

Videos were recorded with a digital videocamera (Sony handycam DCR-PC101E) connected to an external microphone, and edited with Adobe Premiere Pro CS4. Recordings were cut around each verb so that all videos eventually had similar starting and ending points. Each video started just before any movement could be perceived, while the actor still had his arms resting alongside his body, his hands out of the video frame and his mouth closed. Each video ended just after the speech utterance, always during or after the retraction phase of the gesture. Stimuli had an average length of 1.08 s (SD 0.03 s). Corresponding audio-only stimuli were created by masking videos with a black overlay, matched to the background color. The study included 160 different stimuli, all of which were shown to each subject: 40 videos in which speech and gesture were congruent (SGC), the same masked videos so that subjects would only perceive auditory speech from these congruent videos (Sc), 40 videos in which speech and gesture were incongruent (SGI) and the same 40 videos masked (Si). Note that although the sound files in the congruent vs incongruent conditions contained the same words, they came from different videos, hence the distinction between Sc and Si. However, no difference in brain activity was found between these conditions (see results).

**Figure 4:**
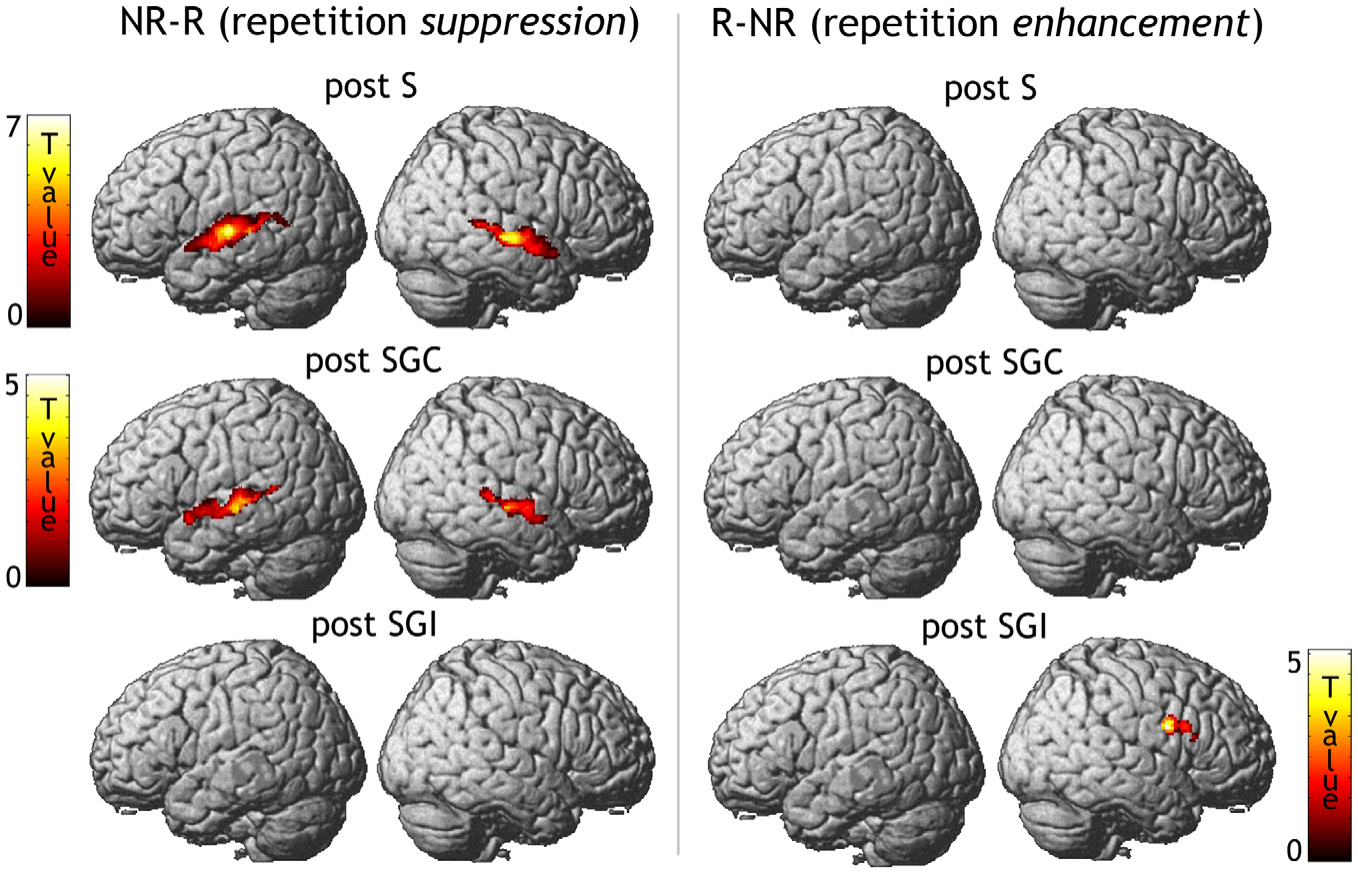
Repetition effects (test phase). SPM renderings of the contrasts NR-R and R-NR following S, SGC, and SGI. Maps were thresholded at p<0.05 corrected for multiple comparisons either in terms of height or spatial extent. See Table 2 for details. R: Repeated, NR: Non-Repeated, S: Speech (average of Si and Sc between which no difference was found), SGC: Congruent Speech and Gesture, SGI: Incongruent Speech and Gesture.

**Figure 5:**
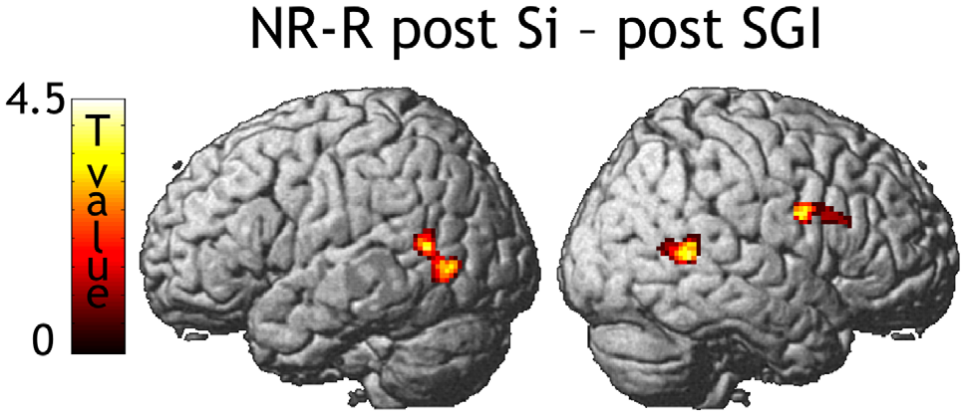
Repetition effects. SPM rendering of the contrast NR-R following S versus SGI. The map was thresholded at p<0.05 corrected for multiple comparisons either in terms of height or spatial extent. The precentral cluster, close to significance (p<0.07 in terms of spatial extent), is shown to illustrate the similarity with the R-NR post SGI contrast shown in figure 4.

**Table 2:** Test phase.

				Test NR-R post					
				S	SGC	SGI	SGC-SGI	SGC-Sc	SGI-Si
Location	*x*	*y*	*z*	Z-score					
L STS	−*66*	−*23*	*4*	**6.3**	**4.2**	2.9	1.3	−0.9	−1.3
R STS	*57*	−*19*	−*1*	**4.9**	**3.4**	3.5	−0.8	0.3	0.4
L STS	−*63*	−*25*	*4*	**5.7**	**4.7**	2.9	1.7	0.3	−1.0
R STS	*53*	−*32*	*6*	**3.5**	**4.5**	1.7	2.4	1.3	−0.4
R precentral G	*53*	*2*	*28*	−0.4	0.2	−**4.8**	3.7	1.6	−4.1
L Precentral G	−*53*	−*4*	*46*	1.9	3.7	−1.9	4.1*	1.4	−1.9
L temporo-parietal	−*55*	−*43*	*27*	1.7	2.8	−2.4	3.9*	2.0	−3.2

Effects in bold are corrected for multiple comparisons either in terms of intensity of spatial extent. Effects marked by a * were Family-Wise Error corrected for multiple comparisons in terms of intensity inside a mask corresponding to brain regions activated by auditory word processing. Coordinates are in the template space of the Montreal Neurological Institute (transformed from the symmetrical template space used for normalization). SGC: Congruent Speech and Gesture, Sc: audio corresponding to SGC, SGI: Incongruent Speech and Gesture, Si: audio corresponding to SGI, NR: Non-Repeated, R: Repeated.

In an effort to characterize gestures used in this study, we asked 8 subjects (4 males/4 females, mean age  = 28 years) to name them. This was done using new videos showing the same actor reenacting the gestures without any speech or mouth movements. Subjects named the gestures using the same action verbs we chose in 38% of the cases. Another 32% of the subjects’ responses were judged to be closely related semantically to the gestures (e.g. “catch” for “grab”, “cross out” for “write” …). Thus, overall, 70% of the responses were in agreement with the meaning we associated to the gestures. This is a reasonable proportion given the non-linguistic nature of the gestures which, contrary to signs, often do not clearly convey any meaning on their own (i.e. without speech).

**Figure 6:**
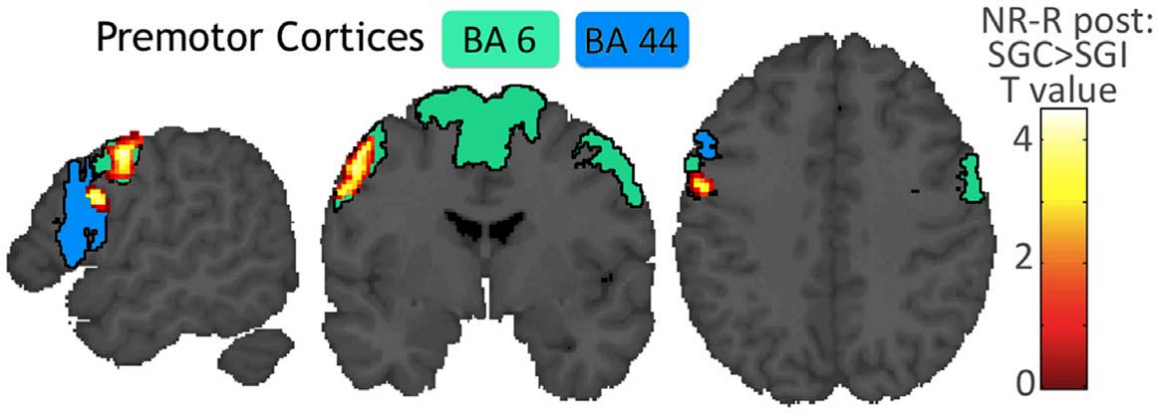
Repetition× Congruence. SPM rendering of the contrast NR-R following SGC versus SGI. Areas colored in blue or green are likely to be located in Brodmann’s cytoarchitectonic area 6 or 44 [37], [38]. The area colored in blue is most likely area 44, the area in green is most likely area 6. The 2 highest intensity peaks in the cluster were located in area 6 with a 100% probability. SGC: Congruent Speech and Gesture, SGI: Incongruent Speech and Gesture, NR: Non-Repeated, R: Repeated.

### fMRI Experiment

#### Setup

Stimuli were presented on a back-projection screen, upon a mirror fastened to a head coil and with the aid of a sound-protecting headset combined with air-conduction headphones. Each video took 14.7° (width) and 11.1° (height) of the visual field. We used Cogent 2000 to display videos and their masked versions (www.vislab.ucl.ac.uk/cogent.php). We checked that subjects could properly perceive an extra video similar to the stimuli during the acquisition of a localizer scan.

**Figure 7:**
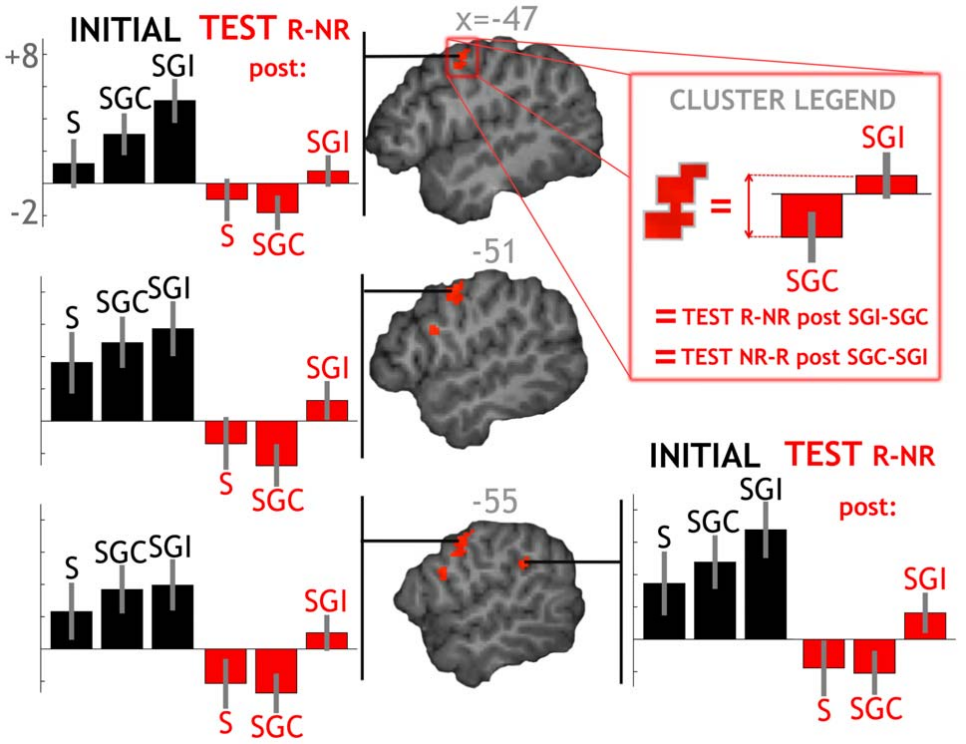
Repetition× Congruence. Plots for the NR-R post SGC-SGI contrast. Two clusters were found after correcting for multiple comparisons inside a mask of auditory effects (see text for details). In each one, plots indicate that repetition suppression was stronger when the word was associated with a congruent iconic gesture. On another hand, repetition enhancement was observed when the word was associated with an incongruent gesture. Full coordinates for these plots in the template space of the Montreal Neurological Institute (transformed from symmetrical template space used for normalization) are as follows. Top: x −47 y −4 z 44; middle: −51 −4 50; bottom left: −55 −4 44; bottom right: −55 −43 27. NR: Non-Repeated, R: Repeated, S: Speech (average of Si and Sc between which no difference was found), SGC: Congruent Speech and Gesture, SGI: Incongruent Speech and Gesture.

#### Sessions and trials

All stimuli were presented to each participant in one fMRI experiment divided in 4 sessions. Stimuli were shown in an event-related manner, using a repetition paradigm where all trials were made of two stimuli of interest - “initial” and “test” - separated by a triple repetition of the initial stimulus (Figure 2). There were 4 types of trials, depending on the nature of the initial stimulus: SGC, Sc, SGI or Si. The inter-stimuli interval was pseudo-randomly varied between 1 and 3 seconds and consisted of the presentation of a fixation cross. There was no interval between the three repeated stimuli in-between the initial and test stimuli. The initial stimulus was either audio or audiovisual whereas the test stimulus was always audio. This test stimulus was either a 5th repetition of the initial stimulus (“R” for “Repeated”) or a non-repeated stimulus, newly presented in the trial (“NR”). Non-repeated stimuli were extracted from the same group of videos as the initial stimulus, i.e. Sc for an initial Sc or SGC, and Si for an initial Si or SGI. Each subject underwent a total of 320 trials  =  [40 SGC trials with repeated test Sc +40 Sc trials with repeated test Sc +40 SGI trials with repeated Si +40 Si trials with repeated Si] + [40 SGC trials with non-repeated test Sc +40 Sc trials with non-repeated test Sc +40 SGI trials with non-repeated Si +40 Si trials with non-repeated Si]. The order of presentation of the 320 trials was pseudo-randomized and specific to each subject. Each of the 4 sessions comprised 80 trials.

**Figure 8:**
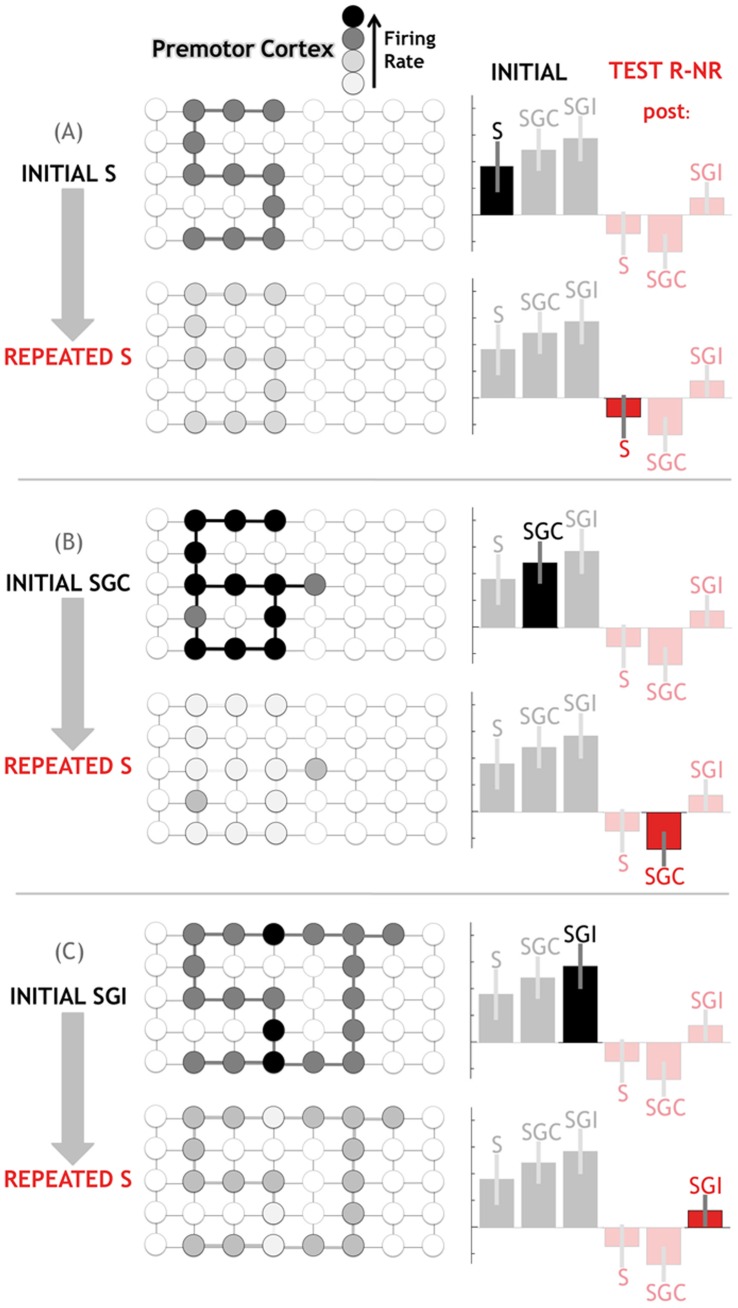
Neuronal model explaining activations and repetition effects associated with the presentation of an action word, and the modulation of theses effects by the audiovisual context. NR: Non-Repeated, R: Repeated, S: Speech (average of Si and Sc between which no difference was found), SGC: Congruent Speech and Gesture, SGI: Incongruent Speech and Gesture.

#### Instructions

Prior to the experiment, subjects were instructed to attentively watch/listen to videos of an actor uttering words. Subjects were warned that the actor would often not be visible - in which case they were asked to continue to pay attention to his speech. There was no mention of gesture. Subjects were also told that they would see a cross at the center of the screen between stimuli indicating that they had nothing to do (“*Rest*” condition). They were not required to fixate the cross. In order to control and encourage subjects’ attention, four trials of questions were added to each session. These trials occurred randomly and rarely (1 out of 20 trials) and consisted in an implicit question given as an instruction to the subject prior to the experiment: “Is the word on the screen the last word you heard?”. Subjects responded by pressing a “yes” or “no” button on a button box (Current Designs, USA) placed under their right-hand. Correct answers were “yes” in half of the cases. Subjects made at most 3 errors/misses out of 16 questions over the entire experiment, and gave on average 90% correct responses. After the experiment, all subjects reported having systematically noticed incongruence between speech and gesture in the audiovisual conditions.

### Image Acquisition

Images were acquired with a Siemens 3T Trio scanner. T2*-weighted echoplanar images with blood oxygenation level-dependent contrast were then acquired in 4 fMRI sessions: 37 3 mm-thick axial slices, no inter-slice interval, 3×3 mm in plane resolution, 64×64 voxels data matrix, FOV 192 mm, TR = 2 s, TE = 30 ms. The TR was not an integer multiple of the Stimulus Onset Asynchrony (SOA = 3.08 s on average), and the SOA was jittered, ensuring distributed sampling of slice acquisition across the experiment [31]. Approximately 530 volumes were acquired per session (depending on variable ISI), leading to a total of 2120 volume images across the 4 sessions. To avoid Nyquist ghost artefacts a generalized reconstruction algorithm was used for data processing. The first three volumes of each session were discarded to allow for T1 equilibration effects. A structural volume was acquired at the end of the fMRI experiment using a high-resolution anatomical T1-weighted 3D sequence: 176 sagittal slices, flip angle  = 9°; Band width  = 150 Hz/pixel, data matrix  = 256 pixel × 256 pixel; pixel size  = 1×1×1 mm (no interslice gap), FOV  = 25.6×25.6 cm, × 17.6 cm in the right left direction, TR  = 2300 ms, TE  = 4.18 ms.

### fMRI Data Analysis

Image processing and statistical analyses were conducted using Statistical Parametric Mapping (SPM8: Wellcome Trust Center for Neuroimaging, London, UK, www.fil.ion.ucl.ac.uk) running under Matlab 7 (Mathworks, Natick, MA, USA).

#### Preprocessing

Each subject´s functional volumes were spatially realigned and unwarped to correct for movement-related signal changes [32]. The normalization was based on the unified segmentation/normalization of the structural image after it had been coregistered to the functional images [33]. The template used was a symmetrical version of the default structural template from the Montreal Neurological Institute implemented in SPM8. This symmetrical template was created by simply copying, flipping along the x axis and averaging the original and the mirror versions of the template. The resulting normalization parameters were then applied to the subject’s functional images thereby rendering them symmetrical. This was relevant to the statistical analyses where we directly compared left and right hemisphere activation by including both flipped and unflipped contrast images - see below [34], [35]. The normalized functional images were then spatially smoothed with a 6 mm full width half maximum isotropic Gaussian kernel to compensate for residual-variability after spatial normalization and to permit application of Gaussian random-field theory for corrected statistical inference [36].

#### Statistical analyses

At the first level, data were analyzed in a subject-specific fashion. All possible event-types were modeled:

Initial SGC, Sc, SGI or Si - from trials where the test audio was repeated or non-repeated (8 event types).
Triplets of the repeated initial stimulus - these were modeled as one event rather than three since there were no interval between the 3 repetitions. Similarly to initial stimuli, triplets were modeled separately for each audiovisual condition: triplets SGC, Sc, SGI, and Si - from trials where the test audio was repeated or non-repeated (8 event types).
Test Sc following SGC, Sc following Sc, Si following SGI, Si following Si - repeated or non-repeated (8 event types).
Questions and button press responses (1 event type).

Overall, 25 (8×3+1) types of events were modeled with the implicit baseline. In order to model each type individually, event-related delta functions were applied to each event and convolved with a hemodynamic response function (HRF). The 25 regressors were then entered in the design matrix and condition effects were estimated along with the general linear model. To exclude low frequency confounds, the data was high-pass filtered using a set of discrete cosine basis functions with a cutoff period of 128 seconds.

Contrasts of interest at the first level were then entered at the second level in 2 sets of ANOVAs to determine:

1. Audio vs. audiovisual effects during the initial presentation of stimuli: contrasts of the different initial types of events vs. fixation (SGC, Sc, SGI and Si from repeated or non-repeated test trials) were entered in a 3×2 ANOVA with factors “modality” (audio vs. audiovisual), “congruence” (congruent vs. incongruent speech and gesture) and “repetition”. The “congruence” factor also applied to audio events because they were extracted from congruent or incongruent videos, allowing one to check for potential indirect effects of speech/gesture congruence on speech perception via a modulation of the actor’s pronunciation (no such effects were found - see results). The “repetition” factor (whether the trial contained a repeated or non-repeated test stimulus) was also modeled.
2. The effect of the prior audiovisual context of repetition on the auditory test phase. This was done in a 2×2 ANOVA in which we entered the [non-repeated test - repeated test] contrast images with “prior modality” and “prior congruence” factors: prior repetition in a congruent audiovisual context (post SGC), prior repetition in an auditory context from congruent videos (post Sc), prior repetition in an incongruent audiovisual context (post SGI) and prior repetition in an auditory context from incongruent videos (post Si). A similar ANOVA was then performed with an additional factor: left vs right, i.e. the symmetrical contrast images were compared to their mirror images to determine whether the repetition*congruence effects were lateralized.

To correct for multiple comparisons we first looked for effects corrected for multiple comparisons across the whole search volume, either in terms of intensity, or in terms of spatial extent (at an uncorrected level of p = 0.001 at the voxel level). Second, we report some of the effects related to the modulation of repetition suppression by the prior audiovisual context (post SGC vs post SGI) inside a mask where there was auditory activation and word-specific auditory repetition suppression effect even at the low threshold of p<0.05 uncorrected (in each case). This mask was independent because auditory activation and auditory repetition effects were measured in trials where there were no audiovisual stimuli. The mask was further constrained by a cluster size threshold of >60 voxels and by the grey matter mask used for normalization.

Cytoarchitectonic localizations were made after bringing functional data into the space of the cytoarchitectonic atlas of the SPM Anatomy toolbox - Jülich, DE [37], [38].

## Results

During the initial phase, posterior temporal, inferior parietal and precentral areas were activated by speech alone (Figure 3 and Table 1). We checked that no difference was observed between activity related to speech Sc extracted from SGC videos and activity related to speech Si extracted from SGI videos (p>0.05 corrected for multiple comparison). Additional occipital, occipito-temporal, inferior parietal and precentral activations were detected in the audiovisual conditions. Critically, no activation was detected in the SGC > SGI contrast even at lower thresholds of significance (p>0.001 uncorrected for multiple comparisons). The reverse contrast revealed more activation in the incongruent condition mainly in parietal and frontal areas. At the behavioral level, incongruence between speech and gesture was reported to be systematically detected.

During the test phase, where words were always presented auditorily, the comparison between new, non-repeated, words (“NR”) with repeated words (“R”) showed repetition suppression bilaterally in the superior part of the temporal lobe, centered on the superior temporal sulcus (STS, Figure 4 and Table 2). However, we only observed this effect when the words had been repeated in an audio-only context (S) or in the congruent speech and gesture audiovisual condition (SGC), but not in the incongruent condition (SGI). In the latter condition, we detected repetition *enhancement* (R>NR) in the right precentral gyrus (Figure 4 and Table 2). This held when controlling for the audio-only condition (R-NR post SGI - post Si  =  NR-R post Si - post SGI, Figure 5 and Table 2). This latter contrast unveiled 2 similar effects bilaterally in the occipito-temporal cortex (Figure 5 and Table 2) where there was a trend for repetition suppression following Si, and a trend for repetition enhancement following SGI.

These effects observed during the test phase suggest that visual information interacts with auditory information during word repetition and that this audiovisual interaction depends on congruency between word and gesture. To address this, we directly compared word repetition effects after the words were repeated in the congruent speech and gesture condition vs after the words were repeated in the incongruent speech and gesture condition ([post SGC NR-R] vs [post SGI NR-R]). We observed a significant effect in the left precentral gyrus (corrected for multiple comparisons in terms of spatial extent), suggesting that the effect localized to the premotor cortex (Figure 6 and Table 2). To confirm this localization, we overlapped the cluster onto probabilistic cytoarchitectonic maps - SPM Anatomy toolbox - [38] of the precentral gyrus. The cluster mainly overlapped with area 6 (54% of the cluster, Figure 6), but also with area 44 (15%) and other areas in the rolandic operculum or not part of cytoarchitectonic maps (31%). One could argue that the decision to use the spatial extent of this cluster as a correction for multiple comparisons was flawed, given that the cluster overlapped with several cytoarchitectonic areas with potentially different functional roles. We therefore focused our search on an independently defined mask of regions, which demonstrated auditory activation and word-specific auditory repetition suppression effects (see methods). By doing so, we confirmed the significance of the effect in left area BA 6 in terms of intensity, and found another significant peak in the left temporo-parietal cortex, which showed a similar pattern of activity across conditions (Figure 7 and Table 2). Furthermore, these differences cannot be attributed to differences between auditory conditions because no difference was observed between the test phase after repetition of speech Sc extracted from SGC videos and the test phase after repetition of speech Si extracted from SGI videos (p>0.05 corrected for multiple comparison). None of these repetition effects were significantly lateralized after correction for multiple comparisons across the whole brain.

Plots in Figure 7 for these areas confirm that there was repetition suppression following repetition of a word either in the audio-only context or in the audiovisual context with a congruent gesture. Plots also show that repetition in the incongruent audiovisual condition led to *enhancement* rather than suppression of the BOLD signal.

Overall, indications that the BOLD signal decreases following repetition of auditory words could only be observed if the words were presented alone or with a congruent gesture, but not with an incongruent gesture.

## Discussion

Our results confirm that speech and audiovisual speech activate perisylvian regions [9], [39], [40], [41], [42], and critically show that co-speech gesture interferes with responses to auditory words in the premotor cortex. Not only did (in)congruency between word and gesture affect premotor activity the first time stimuli were presented, (in)congruency also later affected premotor activity during repetition of the auditory word alone. This straightforward finding supports our hypothesized mechanism for the comparison of speech and gesture. Plots of activity during the various conditions suggest that, following auditory word repetition, premotor activity was reduced only if the word was repeated alone or with a congruent gesture, but not with an incongruent gesture (Figures 7 and 8). According to this mechanism, each word activated a specific premotor representation “S” (for “speech”, Figure 8.A). After repetition of the same word, neurons participating in the representation of this word showed less activity relative to a new, non-repeated word. A significant part of this word representation overlaps with the representation “G” of a gesture associated with the action evoked by the word (Figure 8.B). Therefore after repetition of both word and gesture, activity was even further reduced than after repetition of the word alone. This further repetition may have been caused solely by the mouth movements that were visually perceived alongside gesture. However, this effect disappeared when word and gesture were incongruent, showing the influence of gesture on word processing. We propose that when an incongruent gesture “J” was presented (Figure 8.C), word and gesture representations did not co-localize as well. The same cortical unit showed an even higher BOLD signal in the initial phase because more neurons were activated than in the condition where speech and gesture were congruent. The sum of these neuronal activations outweighed the co-activation of fewer neurons by congruent word and gesture. In the test phase the representation of the auditory word was then associated with many neurons specific to the incongruent gesture. This could explain that the BOLD signal in this phase was even higher than when a new auditory word was presented. In other words, “*repetition enhancement*” here would reflect associative perceptual learning between what subjects recognized to be incongruent word and gesture.

The repetition suppression effects we observed are in agreement with the “fatigue” model of repetition suppression according to which each neuron responding to a given stimulus will also respond less after the same or a similar stimulus is repeated [27], [29]. Our interpretation of the repetition enhancement effects may seem more speculative given the fewer reports of this type of effect and the rare discussions of its potential underlying causes and implications [28], [43], [44]. Yet, studies showing a relation between increased activity and perceptual learning support this account. Gauthier et al. have shown increased activity in the fusiform gyrus of subjects who had developed visual expertise for a novel type of objects [45]. Dolan et al. have also shown increased activation of the fusiform gyrus in subjects who had learned to recognize familiar objects or faces in degraded pictures [46]. Furthermore, Turk-Browne et al. have reported repetition enhancement for low-contrast scenes versus repetition suppression for more easily recognizable high-level contrast scenes [44]. Together, these studies support that repetition enhancement reflects additional processing of initially non-familiar/unrecognized stimuli [47]. We propose that such additional processing also exists in the case of a non-familiar association between word and gesture. This in turn may induce additional processing when the auditory word alone is presented after it has been repeatedly presented with an unexpected gesture.

We further propose that the overlap between representations activated by an action word and an iconic gesture allows for quantifying congruence between these two types of stimuli. This computation would allow the observer to distinguish which of the speaker’s gestures are relevant to his/her discourse. The processing of gesture alone may not be sufficient: the same hand movement may be related or not to discourse. For instance, the speaker may need to briefly scratch his face because it is itching, or the same movement may be an iconic gesture imaging his verbal recall of when he had an itchy beard. A qualitative comparison between speech and gesture, as the one we propose in our model, allows for distinguishing between these two cases.

Additionally, the mechanism we propose could explain why we and others only find more activation for incongruent vs congruent speech and gesture [23], [25]. Holle et al. have reported more activation for congruent vs incongruent speech and gesture in several occipito-temporal, inferior parietal and precentral regions, but the incongruent condition involved hand movements that were not communicative [24]. In addition, these hand movements were systematically directed towards the body (“grooming”). Subjects could therefore understand that these hand movements were not communicative prior to integrating speech and gesture. In another study, Skipper et al. found premotor activity specifically related to gestures congruent with speech, but this was again relative to “grooming” movements unrelated to speech [13]. Although the analysis was of an event-related type, words and gestures were presented to subjects in the context of discourse, and it is unclear whether grooming movements were as synchronized with speech as gestures were. Premotor activity in the congruent condition may therefore have reflected the sum of activities related to speech and gesture, whereas activity during grooming may have been related to hand motion only.

At the macro-anatomical level, premotor and temporo-parietal cortices where we found audiovisual effects are part of the dorsal route for speech processing [5], [6]. In the dorsal route model, the temporo-parietal junction maps auditory information onto articulatory representations stored in premotor cortex. Premotor cortex is also able to represent mouth movements and gesture based on visual input [13], [41], [42]. In line with previous work, we therefore propose that audiovisual integration can take place in premotor cortex because it is a “neutral ground” between the auditory and visual modalities, potentially providing a unifying code in which auditory and visual information pertaining to the same communicative act can be compared [48], [49], [50]. This comparison assumes overlap between representations of the hands and vocal apparatus, a reasonable prerequisite given previous reports of overlapping representations of some body parts and actions in premotor and even primary motor cortex [19], [20], [21], [22]. Finally, the fact that the temporo-parietal junction was sensitive to audiovisual congruence may be explained by a feedback signal from premotor cortex [42].

The dorsal route has been proposed to be an auditory-motor pathway playing a role at the sublexical level [5], [6]. Our results suggest it is also sensitive to visual information of a lexico-semantic nature, in accordance with models where semantic representations involve sensory-motor associations [12]. This also agrees with improved lexical retrieval in normal subjects and Broca’s aphasics when they are allowed or encouraged to gesture [16], [17], [18].

Although our findings point to the dorsal route, it could be argued that they indirectly arise from an integrative process in the ventral route, where auditory word representations are presumably mapped onto semantic representations [5], [6]. We cannot rule out that we missed ventral effects due to reduced signals in temporal regions [51]. Even if this was the case, the lexical status of words may not apply to co-speech gesture. Contrary to words, co-speech gestures are not part of a formal linguistic code, and are not linguistically associated with a specific meaning in an arbitrary way [15], [52]. They are idiosyncratic, specific to each speaker, and hence only interpretable via their iconicity. These characteristics primarily suggest processing by a sensory-motor system similar to the dorsal action perception system, rather than by a lexico-semantic ventral system. On the other hand, while words are linguistic units, they may also be represented as articulatory gestures in a dorsal sensory-motor system, which therefore turns out to be the best candidate for the comparison of gesture and speech. Note that we do not claim that the ventral route for speech processing plays no role in integrating speech and gesture. Rather, the mechanism suggested by our data does not require it. In the context of discourse, Skipper et al. found stronger connectivity between the precentral gyrus and anterior temporal areas in a congruent speech and gesture condition [13] suggesting that gesture influences semantic processes taking place in temporal areas of the ventral route [10], [13], [39], [53]. Therefore, a more accurate model of speech and iconic gesture integration may include both the dorsal and the ventral route, starting with the dorsal route - where the comparison between single words and iconic gestures takes place, which then influences the ventral route, most prominently at the sentence and discourse levels.

In summary, our data suggest that auditory and visual information from speech and gesture converge onto the premotor cortex of the dorsal route for speech processing where it is conveniently represented as unimodal information. Information from speech and gesture could thus be compared, and (in)congruence could be inferred from the size of the overlap between the populations of sensory-motor neurons activated by speech and gesture respectively, with congruence leading to a large overlap, and incongruence leading to a small or no overlap.

## References

[1] Dax M (1865) Lesions de la moitie gauche de l’encephale coincidant avec l’oubli des signes de la pensee (Lu au Congres Meridional tenu a Montpellier en 1836. Gazette Hebdomadaire de Medecine et de Chirurgie 2: 259–260.

[2] Hecaen H, De Agostini M, Monzon-Montes A (1981) Cerebral organization in left-handers. Brain Lang 12: 261–284. 7214131 10.1016/0093-934x(81)90018-3

[3] Haxby JV, Grady CL, Horwitz B, Ungerleider LG, Mishkin M, et al (1991) Dissociation of object and spatial visual processing pathways in human extrastriate cortex. Proc Natl Acad Sci U S A 88: 1621–1625. 2000370 10.1073/pnas.88.5.1621 PMC51076

[4] Alain C, Arnott SR, Hevenor S, Graham S, Grady CL (2001) “What” and “where” in the human auditory system. Proc Natl Acad Sci U S A 98: 12301–12306. 11572938 10.1073/pnas.211209098 PMC59809

[5] Hickok G, Poeppel D (2004) Dorsal and ventral streams: a framework for understanding aspects of the functional anatomy of language. Cognition 92: 67–99. 15037127 10.1016/j.cognition.2003.10.011

[6] Hickok G, Poeppel D (2007) The cortical organization of speech processing. Nat Rev Neurosci 8: 393–402. 17431404 10.1038/nrn2113

[7] Griffiths TD (2008) Sensory systems: auditory action streams? Curr Biol 18: R387–388. 18460320 10.1016/j.cub.2008.03.007

[8] Liberman AM, Cooper FS, Shankweiler DP, Studdert-Kennedy M (1967) Perception of the speech code. Psychol Rev 74: 431–461. 4170865 10.1037/h0020279

[9] Hickok G, Buchsbaum B, Humphries C, Muftuler T (2003) Auditory-motor interaction revealed by fMRI: speech, music, and working memory in area Spt. J Cogn Neurosci 15: 673–682. 12965041 10.1162/089892903322307393

[10] Price CJ (2010) The anatomy of language: A review of 100 fMRI studies published in 2009. Annals of the New York Academy of Sciences. In press. 10.1111/j.1749-6632.2010.05444.x 20392276

[11] Hauk O, Johnsrude I, Pulvermuller F (2004) Somatotopic representation of action words in human motor and premotor cortex. Neuron 41: 301–307. 14741110 10.1016/s0896-6273(03)00838-9

[12] Pulvermuller F, Fadiga L (2010) Active perception: sensorimotor circuits as a cortical basis for language. Nat Rev Neurosci 11: 351–360. 20383203 10.1038/nrn2811

[13] Skipper JI, Goldin-Meadow S, Nusbaum HC, Small SL (2009) Gestures orchestrate brain networks for language understanding. Curr Biol 19: 661–667. 19327997 10.1016/j.cub.2009.02.051 PMC3767135

[14] Kendon A (2004) Gesture : visible action as utterance. Cambridge: Cambridge University Press.

[15] McNeill D (1992) Hand and mind : what gestures reveal about thought: University of Chicago Press.

[16] Rose M, Douglas J (2001) The differential facilitatory effects of gesture and visualisation processes on object naming in aphasia. Aphasiology 15: 977–990.

[17] Rose M, Douglas J, Matyas T (2002) The comparative effectiveness of gesture and verbal treatments for a specific phonologic naming impairment. Aphasiology 16: 1001–1030.

[18] Krauss R (1998) Why do we gesture when we speak? Current Directions in Psychological Science 7: 54–59.

[19] Gentilucci M, Benuzzi F, Gangitano M, Grimaldi S (2001) Grasp with hand and mouth: a kinematic study on healthy subjects. J Neurophysiol 86: 1685–1699. 11600632 10.1152/jn.2001.86.4.1685

[20] Graziano M (2006) The organization of behavioral repertoire in motor cortex. Annu Rev Neurosci 29: 105–134. 16776581 10.1146/annurev.neuro.29.051605.112924

[21] Schieber MH (2001) Constraints on somatotopic organization in the primary motor cortex. J Neurophysiol 86: 2125–2143. 11698506 10.1152/jn.2001.86.5.2125

[22] Meister IG, Buelte D, Staedtgen M, Boroojerdi B, Sparing R (2009) The dorsal premotor cortex orchestrates concurrent speech and fingertapping movements. Eur J Neurosci 29: 2074–2082. 19453637 10.1111/j.1460-9568.2009.06729.x

[23] Dick AS, Goldin-Meadow S, Hasson U, Skipper JI, Small SL (2009) Co-speech gestures influence neural activity in brain regions associated with processing semantic information. Hum Brain Mapp. 10.1002/hbm.20774 PMC2896896 19384890

[24] Holle H, Gunter TC, Ruschemeyer SA, Hennenlotter A, Iacoboni M (2008) Neural correlates of the processing of co-speech gestures. Neuroimage 39: 2010–2024. 18093845 10.1016/j.neuroimage.2007.10.055

[25] Willems RM, Ozyurek A, Hagoort P (2009) Differential roles for left inferior frontal and superior temporal cortex in multimodal integration of action and language. Neuroimage 47: 1992–2004. 19497376 10.1016/j.neuroimage.2009.05.066

[26] Willems RM, Ozyurek A, Hagoort P (2007) When language meets action: the neural integration of gesture and speech. Cereb Cortex 17: 2322–2333. 17159232 10.1093/cercor/bhl141

[27] Sawamura H, Orban GA, Vogels R (2006) Selectivity of neuronal adaptation does not match response selectivity: a single-cell study of the FMRI adaptation paradigm. Neuron 49: 307–318. 16423703 10.1016/j.neuron.2005.11.028

[28] Grill-Spector K (2006) Selectivity of adaptation in single units: implications for FMRI experiments. Neuron 49: 170–171. 16423690 10.1016/j.neuron.2006.01.004

[29] Grill-Spector K, Henson R, Martin A (2006) Repetition and the brain: neural models of stimulus-specific effects. Trends Cogn Sci 10: 14–23. 16321563 10.1016/j.tics.2005.11.006

[30] Oldfield RC (1971) The assessment and analysis of handedness: the Edinburgh inventory. Neuropsychologia 9: 97–113. 5146491 10.1016/0028-3932(71)90067-4

[31] Veltman DJ, Mechelli A, Friston KJ, Price CJ (2002) The importance of distributed sampling in blocked functional magnetic resonance imaging designs. Neuroimage 17: 1203–1206. 12414260 10.1006/nimg.2002.1242

[32] Andersson JL, Hutton C, Ashburner J, Turner R, Friston K (2001) Modeling geometric deformations in EPI time series. Neuroimage 13: 903–919. 11304086 10.1006/nimg.2001.0746

[33] Ashburner J, Friston KJ (2005) Unified segmentation. Neuroimage 26: 839–851. 15955494 10.1016/j.neuroimage.2005.02.018

[34] Jancke L, Wustenberg T, Scheich H, Heinze HJ (2002) Phonetic perception and the temporal cortex. Neuroimage 15: 733–746. 11906217 10.1006/nimg.2001.1027

[35] Baciu M, Juphard A, Cousin E, Bas JF (2005) Evaluating fMRI methods for assessing hemispheric language dominance in healthy subjects. Eur J Radiol 55: 209–218. 16036149 10.1016/j.ejrad.2004.11.004

[36] Friston KJ, Holmes AP, Poline JB, Grasby PJ, Williams SC, et al (1995) Analysis of fMRI time-series revisited. Neuroimage 2: 45–53. 9343589 10.1006/nimg.1995.1007

[37] Eickhoff SB, Paus T, Caspers S, Grosbras MH, Evans AC, et al (2007) Assignment of functional activations to probabilistic cytoarchitectonic areas revisited. Neuroimage 36: 511–521. 17499520 10.1016/j.neuroimage.2007.03.060

[38] Eickhoff SB, Stephan KE, Mohlberg H, Grefkes C, Fink GR, et al (2005) A new SPM toolbox for combining probabilistic cytoarchitectonic maps and functional imaging data. Neuroimage 25: 1325–1335. 15850749 10.1016/j.neuroimage.2004.12.034

[39] Mazoyer B, Dehaene S, Tzourio N, Murayama N, Cohen L, et al (1993) The cortical representation of speech. Journal of Cognitive Neuroscience 5: 467–479. 23964919 10.1162/jocn.1993.5.4.467

[40] Wilson SM, Saygin AP, Sereno MI, Iacoboni M (2004) Listening to speech activates motor areas involved in speech production. Nat Neurosci 7: 701–702. 15184903 10.1038/nn1263

[41] Skipper JI, Nusbaum HC, Small SL (2005) Listening to talking faces: motor cortical activation during speech perception. Neuroimage 25: 76–89. 15734345 10.1016/j.neuroimage.2004.11.006

[42] Skipper JI, van Wassenhove V, Nusbaum HC, Small SL (2007) Hearing lips and seeing voices: how cortical areas supporting speech production mediate audiovisual speech perception. Cereb Cortex 17: 2387–2399. 17218482 10.1093/cercor/bhl147 PMC2896890

[43] Henson RN, Rugg MD (2003) Neural response suppression, haemodynamic repetition effects, and behavioural priming. Neuropsychologia 41: 263–270. 12457752 10.1016/s0028-3932(02)00159-8

[44] Turk-Browne NB, Yi DJ, Leber AB, Chun MM (2007) Visual quality determines the direction of neural repetition effects. Cereb Cortex 17: 425–433. 16565294 10.1093/cercor/bhj159

[45] Gauthier I, Tarr MJ, Anderson AW, Skudlarski P, Gore JC (1999) Activation of the middle fusiform ‘face area’ increases with expertise in recognizing novel objects. Nat Neurosci 2: 568–573. 10448223 10.1038/9224

[46] Dolan RJ, Fink GR, Rolls E, Booth M, Holmes A, et al (1997) How the brain learns to see objects and faces in an impoverished context. Nature 389: 596–599. 9335498 10.1038/39309

[47] Henson RN (2003) Neuroimaging studies of priming. Prog Neurobiol 70: 53–81. 12927334 10.1016/s0301-0082(03)00086-8

[48] Kohler E, Keysers C, Umilta MA, Fogassi L, Gallese V, et al (2002) Hearing sounds, understanding actions: action representation in mirror neurons. Science 297: 846–848. 12161656 10.1126/science.1070311

[49] Driver J, Noesselt T (2008) Multisensory interplay reveals crossmodal influences on ‘sensory-specific’ brain regions, neural responses, and judgments. Neuron 57: 11–23. 18184561 10.1016/j.neuron.2007.12.013 PMC2427054

[50] Kaplan JT, Iacoboni M (2007) Multimodal action representation in human left ventral premotor cortex. Cogn Process 8: 103–113. 17503101 10.1007/s10339-007-0165-z

[51] Devlin JT, Russell RP, Davis MH, Price CJ, Wilson J, et al (2000) Susceptibility-induced loss of signal: comparing PET and fMRI on a semantic task. Neuroimage 11: 589–600. 10860788 10.1006/nimg.2000.0595

[52] McNeill D (2005) Gesture and Thought: University of Chicago Press.

[53] Demonet JF, Thierry G, Cardebat D (2005) Renewal of the neurophysiology of language: functional neuroimaging. Physiol Rev 85: 49–95. 15618478 10.1152/physrev.00049.2003

